# Dual graph characteristics of water distribution networks—how optimal are design solutions?

**DOI:** 10.1007/s40747-022-00797-4

**Published:** 2022-06-23

**Authors:** Robert Sitzenfrei, Mohsen Hajibabaei, Sina Hesarkazzazi, Kegong Diao

**Affiliations:** 1 Faculty of Engineering Sciences, Department of Infrastructure Engineering, University Innsbruck, Unit of Environmental Engineering, Technikerstrasse 13, Innsbruck, Austria; 2 Faculty of Computing, Engineering, and Media, De Montfort University, The Gateway, Leicester, LE1 9BH UK

**Keywords:** Optimization, Multi-objective, Dual mapping, Hierarchical intersection continuity negotiation, Demand edge betweenness centrality

## Abstract

Urban water infrastructures are an essential part of urban areas. For their construction and maintenance, major investments are required to ensure an efficient and reliable function. Vital parts of the urban water infrastructures are water distribution networks (WDNs), which transport water from the production (sources) to the spatially distributed consumers (sinks). To minimize the costs and at the same time maximize the resilience of such a system, multi-objective optimization procedures (e.g., meta-heuristic searches) are performed. Assessing the hydraulic behavior of WDNs in such an optimization procedure is no trivial task and is computationally demanding. Further, deciding how close to optimal design solutions the current solutions are, is difficult to assess and often results in an unnecessary extent of experiment. To tackle these challenges, an answer to the questions is sought: when is an optimization stage achieved from which no further improvements can be expected, and how can that be assessed? It was found that graph characteristics based on complex network theory (number of dual graph elements) converge towards a certain threshold with increasing number of generations. Furthermore, a novel method based on network topology and the demand distribution in WDNs, specifically based on changes in ‘demand edge betweenness centrality’, for identifying that threshold is developed and successfully tested. With the proposed novel approach, it is feasible, prior to the optimization, to determine characteristics that optimal design solutions should fulfill, and thereafter, test them during the optimization process. Therewith, numerous simulation runs of meta-heuristic search engines can be avoided.

## Introduction

Urban water infrastructures are an essential part of urban areas. For their construction and maintenance, major investments are required, to ensure an efficient and reliable function. Vital parts of urban water infrastructures are the water distribution networks (WDNs), in which water is transported from the production (sources) to the spatial distributed consumers (sinks). The hydraulics of WDNs follow physical principles (i.e., conservation of mass and energy) but determining this hydraulic behavior is not a trivial task; therefore, WDNs are categorized as complex networks [1].

A WDN should provide potable water of sufficient quantity and quality to consumers [2]. Due to the high costs of construction and maintenance, design has to be economically viable and within the defined budget. Besides costs, additional performance metrics, such as resilience, reliability, leakage, or water quality, are also crucial for these systems, making the WDN design a multi-objective optimization problem [2]. In the optimal design process of WDNs, pipe diameters have to be chosen while at least a minimum head needs to be maintained at all nodes. Water pipes are manufactured in a discrete set of pipe diameters, which introduces significant difficulties in solving such a problem [3]. This is due to the discontinuous, highly nonlinear, constrained and multi-modal search space [4] introducing NP (non-deterministic polynomial-time) hard characteristics [5], which can be at least approximated with e.g., meta-heuristics but require expensive numerical simulations [6]. Specifically, for large-scale problems, finding a trade-off between exploration and exploitation is important to avoid unnecessary experiments. Therefore, different learning strategies [7], surrogate-assisted evolutionary algorithms [8], or decomposition of the problem formulation [9] can be applied while ensuring the diversity of the populations. There is manifold literature on the optimal design of WDNs [10–12]. The problem formulation in this regard can be quite diverse as the objective functions can be chosen very differently (e.g., minimizing costs, maximizing performance or robustness, etc.). To assess the performance (i.e., the objective function) of different solutions, the transport processes (i.e., hydraulics) in WDNs must be assessed with hydraulic solvers. For the simulation of different system states of WDNs with such hydraulic solvers, a set of nonlinear equations has to be solved multiple times for each design candidate [13]. Specifically for large-scale problems, this procedure requires a significant amount of computational time [2]. To save computational time, often when using meta-heuristics for multi-objective optimization, stopping criteria (which is commonly a heuristic itself), are used. The stopping criterion for population-based methods is often the number of generations or the number of fitness function evaluations. The choice of these values depends on the complexity of WDNs and the number of decision variables [14]. Also, convergence criteria can be used, evaluating, e.g., if the best-ranked individuals do not change over several generations [15]. However, with increasing complexity of WDNs also the computational efforts increase and available computational budgets do not allow to satisfactorily achieve such a state and such an evaluation might have significant computational overheads [16]. Further, the discrete decision space for diameter choices might result in back and forth change of diameters, making it difficult to assess convergence. At the start, it remains unclear where this stopping criterion might be. When looking at the spatial characteristics of optimal designs (i.e., the sequence of pipe diameters and diameter changes), specific patterns can be identified throughout a Pareto front of optimal design solutions [17] (i.e., diameter changes mostly occur only when there are demand changes). Better understanding such characteristics could help to decide whether the optimization process should be continued or not.

A WDN can be represented as a mathematical graph in which specific network patterns are reoccurring [18, 19]. From a network perspective, a multi-objective optimization (e.g., costs versus resilience) is of great interest, providing a set of Pareto-optimal solutions for decision making. Sitzenfrei et al. [20] identified graph characteristics of WDNs, which were optimized with an evolutionary optimization approach by minimizing the costs while maximizing the resilience. In Jiang and Claramunt [21], the vertices of the dual graph represented named streets, and the dual links represented street intersections. Rosvall et al. [22] interpreted the dual characteristics as information space for navigation, i.e., how much information is needed to navigate from one point to another. Porta et al. [23] suggested using a generalization model to reduce complexity named Intersection Continuity Negotiation, enabling a continuation of street sections by determining the geometrical angles between the edges with the highest continuity. They further investigated the structural properties of the determined dual graph of the street network. Masucci et al. [24] investigated the London street network with a hybrid approach, combining the information space with geometrical attributes (angels) and road classes (Hierarchical Intersection Continuity Negotiation—HICN). Zischg et al. [25] investigated the topological coevolution of three different urban networks (i.e., water distribution, drainage, and street network) based on the HICN approach to create the dual graphs. They showed that angles between the edges of WDNs do not play a crucial role when investigating the dual characteristics. They suggested using the edge diameter as a functional entity for the HICN approach in WDNs, as it is a surrogate measure for pipe capacity.

For gaining a deeper insight into the functional properties of optimal transport networks and also the progress of the optimization process, a dual graph approach is missing in literature. While primal graph approach usually represents the geographic dimension of a spatial network, dual approaches reduce the complexity of a network by aggregating identical information like pipe diameters and therefore reducing the search space for meta-heuristic optimization algorithms. This study aims to address the research gap of stopping criterion in meta-heuristic optimization by developing a dual graph approach for describing the network characteristics during the optimization process. Therefore, the dual characteristics of Pareto-optimal WDNs for three cases, including the pathway for optimization from random initialization to Pareto-optimal solutions, are investigated. Subsequently, the dual characteristics of these WDNs are systematically investigated to identify properties of (partly) optimal WDNs and to verify whether dual characteristics can be used as an indicator of how close from optimal solutions are. By that an answer to the questions is sought: when is a sufficient optimization stage achieved and how can that be assessed? Further, a generic estimation based on demand edge betweenness centrality is identified to give a meaningful measure of whether an evolutionary algorithm is close to the optimal solution, and an estimation of the necessary progress in the optimization process is given.

## Materials and methods

In this section, first, an overview of optimization of WDNs is given. It is only an overview, as the proposed method for assessing the progress of the optimization is a generic approach, which can be used with any kind of meta-heuristic optimization approaches. Further details on optimization can be found in recent literature [10, 26, 27]. Subsequently, the graph measures used in this work are shown. Then as a significant step in this work, the primal and dual graph creation are discussed in detail with a step by step description of the proposed approach. Finally, real case studies used to show the applicability of the proposed approach are introduced, including the Pareto-fronts of optimal design solutions (including the different generations).

### Design and multi-objective optimization of WDNs

A WDN consists of several different components which transport drinking water from the origin of its production (wells, reservoirs, springs, etc.) to the location of its consumption (e.g., domestic, commercial or industrial demand nodes). For the transport process, elements like pipes, valves and pumps are used. These transport elements usually cause the major part of investment and maintenance costs in a WDN and therefore the optimal design of these is of great relevance [20]. Minimizing costs for the pipe design in this regard is a very important objective but results in just sufficient performance for the design load. However, the supply of water needs to be reliable and should be able to cope also with unexpected and/or critical conditions. Therefore, objectives like resilience or robustness of WDNs are also considered in the design, which conflicts the least cost objective. Therefore, the design task is intrinsically a multi-objective optimization problem [28] which results in a Pareto front of optimal designs. The multi-objective design problem of WDNs is often tackled by meta-heuristic methods [27] which are cumbersome to solve. This is due to its large number of decision variables (e.g., number of pipes) and discrete decision space (available discrete classes for pipe diameters), which is especially the case for large complex WDNs. Many researchers focused on the “end-of-run” performance of meta-heuristics (quality of the final Pareto front), considering the multi-objective optimization as a black box model. However less attention was given to the evolutions of the solutions during the optimization process [29]. This work aims to explore the evolution of the solutions in the optimization process and to find a metric assisting the process. Therefore, in this work, for the pipe design (diameter choices) the state-of-the-art methodology GALAXY (Genetically Adaptive Leaping Algorithm for approXimation and diversitY) is used, which is based on multi-objective evolutionary algorithms [26] such as the NSGA-II approach [30]. The designs obtained from GALAXY are subsequently analyzed with different customized graph analysis (see chapter Graph measures for WDNs). Note that for the presented methodology based on complex network theory, any kind of evolutionary algorithm can be used without changing the presented generic approach.

In GALAXY, a random initial population for the pipe designs is generated within the variable domains (available discrete pipe diameters) and subsequent, the objective functions are evaluated. Subsequently, individuals in the population are ranked using a non-dominated sorting procedure [30]. Based on the best ranked individuals (designs) an offspring generation is created with search operators. With a replacement strategy it is determined which elements of the designs are passed on to the next generation. This procedure is usually repeated until the predefined number of generations is obtained. Because of the discrete structure of the decision variables space (restricted to integers), it is important that search operators can cope with a “leaping” in search space [26]. Therefore, different search operators are implemented, which work simultaneously to ensure a good balance between exploration and exploitation. In GALAXY as search operators, Turbulence Factor, Differential Evolution, Simulated Binary Crossover for Integers, Uniform Mutation, and Dither Creeping are used as these ensure a wide range of applicability for the optimization of WDNs [26]. A hybrid replacement strategy is used in the replacement process, specifically the Pareto-dominance [30] in combination with the ε-dominance concept [31].

Two conflicting objectives, i.e., minimizing costs and maximizing resilience, are used for optimization resulting in a Pareto front of design solutions. The total costs are calculated based on the unit pipe costs as a function of discrete pipe diameters and pipe lengths [20].

For a reliable supply, water demands at demand nodes have to be ensured, resulting in minimal head requirement (the supplied demand is a function of the available head [32]). Therefore, to determine the available heads in WDNs, the governing equations of the transport network (conservation of mass and energy) are solved. For this purpose, the state-of-the-art hydraulic solver is Epanet2 is used in this study [33]. A reliable supply can be described with the network resilience. The network resilience (*I*_r_) in this work is determined according to Prasad and Park [34] as a single metric for each design solution, calculated between − ∞ (poor) and 1 (best). Therein, the output from the system (counter in Eq. 1), is divided by sum over the inputs to the system minus the minimum required output (denominator in Eq. 1). The output is determined by the sum over the number of nodes #*N* of the product of nodal surplus head (*H*_*j*_ − *H*_min_), uniformity of pipe connections to a node (*C*_*j*_) and nodal demands (*Q*_*j*_). The sum over the inputs is determined by the sum over the number of reservoirs #S, reservoir flows (*Q*_*k*_) and heads (*H*_*k*_) and pump powers *P*_*i*_/γ and the minimum required input equals to sum of nodal demands *Q*_*j*_ times minimal head (*H*_min_). *I*_r_ is determined by Eq. (1) as follow:

1 $${I}_{\mathrm{r}}=\frac{\sum_{j=1}^{\#N}{C}_{j}\cdot {Q}_{j}\cdot \left({H}_{j}-{H}_{min}\right)}{\left[{\sum }_{k=1}^{\#S}{Q}_{k}\cdot {H}_{k}+{\sum }_{i=1}^{\#P}{P}_{i}/\gamma \right]-{\sum }_{j=1}^{\#N}{Q}_{j}\cdot {H}_{min}}$$

The uniformity *C*_*j*_ of a node *j* to which *npj* pipes are connected with the diameters *d*_*i*_ is determined with:

2 $${C}_{{j}}\text{=}\frac{{\sum }_{{{i}}= {1} }^{{npj}}{{{d}}}_{{i}}}{{{npj}}\cdot{\max(}{{d}}_{{i}}{)}}$$

### Graph measures for WDNs

A graph is a mathematical representation of a network, consisting of a set of vertices (nodes) which are interconnected via edges (pipes). WDNs can be represented in a simplified way by graphs. The advantage of using graphs instead of e.g., hydraulic analysing is that graph analyses require much less computational efforts (which comes with some loss of accuracy) [2]. Therefore, graph measures can be useful for different tasks in WDNs [35, 36]. In this work, the graph measures called demand edge betweenness centrality (*d*_EBC_) and mean/average node degree (nd) are applied. The node degree of a network represents the number of edges connected to a node [37]. The average node degree *nd* can be calculated with the total number of edges #*E* and the total number of nodes #*N*:

3 $$nd=\frac{2\cdot \#E}{\#N}$$

*d*_EBC_ is a customized graph measure defined for WDNs based on edge betweenness centrality EBC [38]. EBC(*k*) values for an edge *k* measure how often that edge is part of a shortest path σ_*ij*_ between all node pairs *i* and *j* [20]. That measure can be tailored considering the complexity of WDNs, e.g., for source nodes *i*, the water intakes (e.g., tanks, reservoirs, or wells), and for target nodes *j*, the demand nodes *D* are used. Further, instead of counting the number of shortest paths passing through an edge between *i* and *j*, the demand *Q*_*j*_ of the target nodes *j* is summed up resulting in the Eq. (4) as follow:

4 $$ \begin{aligned} \mathrm{EBC}\left(k\right) & =\sum_{i, j\in D}{\sigma }_{i,j}\left(k\right)\cdot {Q}_{j}\,\mathrm{with\, EBC}\left(k\right)\;\mathrm{in\,the\,limits\,of }\\ &\quad \left[0 \sum_{j\in D}{Q}_{j}\right] \end{aligned}$$

The EBC(*k*) values of the edges can then be grouped according to design flows of different diameters (assuming a flow velocity of e.g., 0.5 m/s, applying continuity equation and rounding to the next available larger discrete diameter) [20]. The number of groups indicates how many diameter changes are necessary based on the demand distribution, denoted d_EBC_. The d_EBC_ values could also be used for the design itself; however, such an approach results in a small portion of the solutions of the entire Pareto front [39].

### Primal graph representation

Besides a representation in a geographical information system, a common format for the graph of a WDN is the input for the hydraulic model. There are rarely topological errors in hydraulic models due to the functional (hydraulic) verification. The state-of-the-art platform for the hydraulic modelling is Epanet 2 [33]. In this work, an interface from Epanet 2 to Matlab is used, which was developed by Sitzenfrei, Oberascher [17]. The interface also provides simulated parameters (flows, head loss, flow directions, water quality, etc.) as additional graph properties, which can be used for graph weights, attributes or directions.

### Dual graph representation

**Fig. 1:**
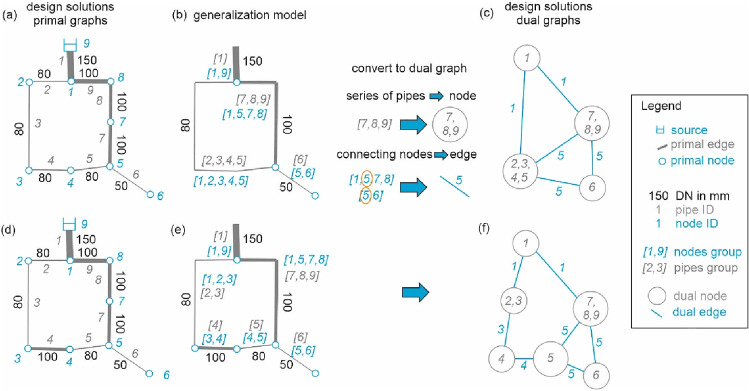
Dual graph creation procedure

To reduce the complexity of a primal graph, generalization models can be used, which aggregate elements with identical characteristics. In an optimal WDNs, often identical diameters are clustered [17]. To use this characteristic in meta-heuristic optimization algorithms, in this work, a dual graph creation procedure specifically tailored for WDNs is developed in Matlab. As suggested by Zischg et al. [25], the edge diameter *d*_*k*_ as a functional entity is used as a generalization model as a surrogate measure for capacity driving the costs and the performance. The procedure uses the primal graph of a WDN (including also hydraulic properties) as input, outlined as follows:

1. The procedure starts with a random edge *e*_*k*_, with a diameter *d*_*i*_, chosen from a set of edges from the primal graph. The status of all other edges is set to “not found”.
2. Subgraphs are created, containing only edges with diameters equal to the current *d*_*k*_ of interest and edges with a status “not found”.
3. From the start edge *e*_*k*_, a breadth-first search [40] is performed on the subgraphs identifying all edges connected to *e*_*k*_ (and therefore have also the same diameter *d*_*k*_). Alternatively, a connected component analysis could have also been performed, but the breadth-first search was implemented to have a generic approach, also for other generalization models.
4. All identified edges from step 3 and their start and end nodes are generalized and integrated into a new dual node. The status of the found edges is subsequently set to “found” in the primal graph.
5. The next start edge *e*_*k*+1_ is randomly chosen from the edges of the primal graph with the status “not found” and the associated diameter *d*_*k*+1_ is determined.
6. Steps 2 to 5 are repeated until all edges’ status is “found”, and therefore, the set of all dual nodes is created.
7. Primal nodes which are part of multiple dual nodes are identified (e.g., orange marked in Fig. 1); these are then the dual edges connecting these dual nodes.
8. A dual graph with a set of dual nodes and dual edges is created.

In Fig. 1, a toy example of the proposed dual graph creation from the primal WDN graph for two different design solutions (a) and (d) is shown. It can be seen how a difference in the diameter in pipe 4 leads to different dual graph configurations (Fig. 1c, f).

The dual graphs of different design solutions from the Pareto front and the optimization pathway are subsequently analyzed regarding the number of dual nodes and average node degrees nd. The number of dual nodes is analyzed for different generations but in one generation, there is a multitude of design solutions (equal to the population size). Further, there are several hundred thousand generations for each case study (see description of case studies). Therefore, for a clearly arranged systematic analysis, a subset of generations is analyzed (e.g., 1st, 10th, 100th 1000th, …) and then statistical values of an entire generation are used (e.g., median values, 25% and 75% percentiles).

### Real world case studies

**Fig. 2:**
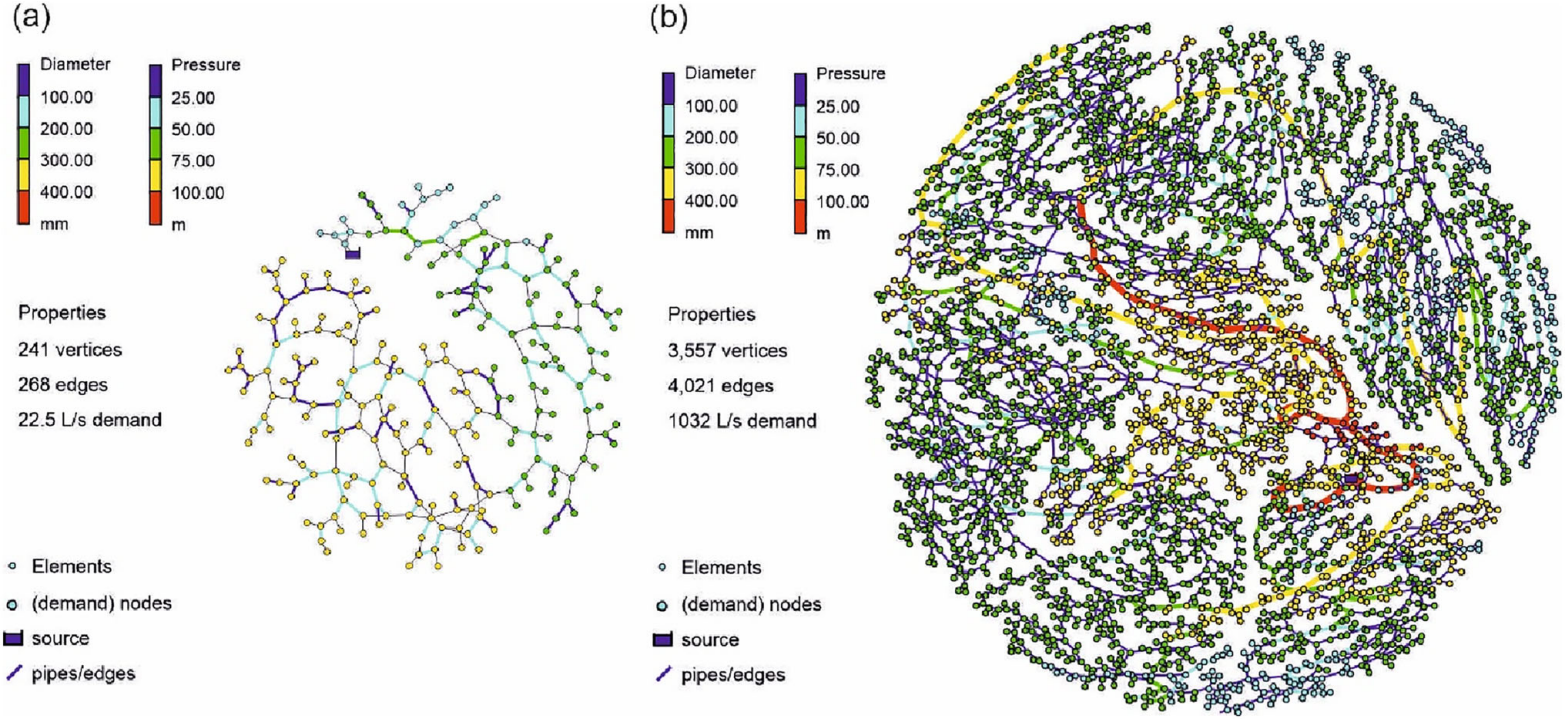
Force-directed placement of the investigated real WDNs

WDNs are crucial for human well-being and also the functioning of modern society. Therefore, they belong to critical infrastructures which are specifically highly protected [41]. Consequently, the real spatial layout of the investigated WDN cannot be shown here, respectively, it needs to be anonymized. Instead of showing the graphs in the Euclidean space, an anonymized graph drawing by force-directed placement [42] is used, which aims to make edge lengths uniform while minimizing the edge crossings. However, the hydraulics of the real WDNs are fully preserved by this procedure and all following results are based on the hydraulics of the real WDNs. The first graph (see Fig. 2a) is a WDN of a real case study with 242 vertices and 268 edges with a mean node degree *nd* of 2.21. It has 118 demand nodes, with a total demand of 22.5 L/s. The total network length is 14.4 km and the average edge length is 53.96 m. It has one source which supplies the entire WDN fully gravity-driven. It is assumed, that the spatial demand distribution has a major impact on the characteristics of the optimized WDNs. Therefore, to investigate how these demand variations would affect the characteristics of the dual graphs, an additional scenario is considered, wherein the aforementioned WDN is assigned with only ten demand nodes with the same total demand of 22.5 L/s. The ten demand nodes are distributed manually to spatially cover the entire supply area.

The graph of the large investigated real WDN has 3558 vertices and 4021 edges with a mean node degree of 2.26. The network length is 211 km and the average edge length is 52.68 m. It has one source node, which supplies the entire WDN, with a total demand of 1131.78 L/s.

**Fig. 3:**
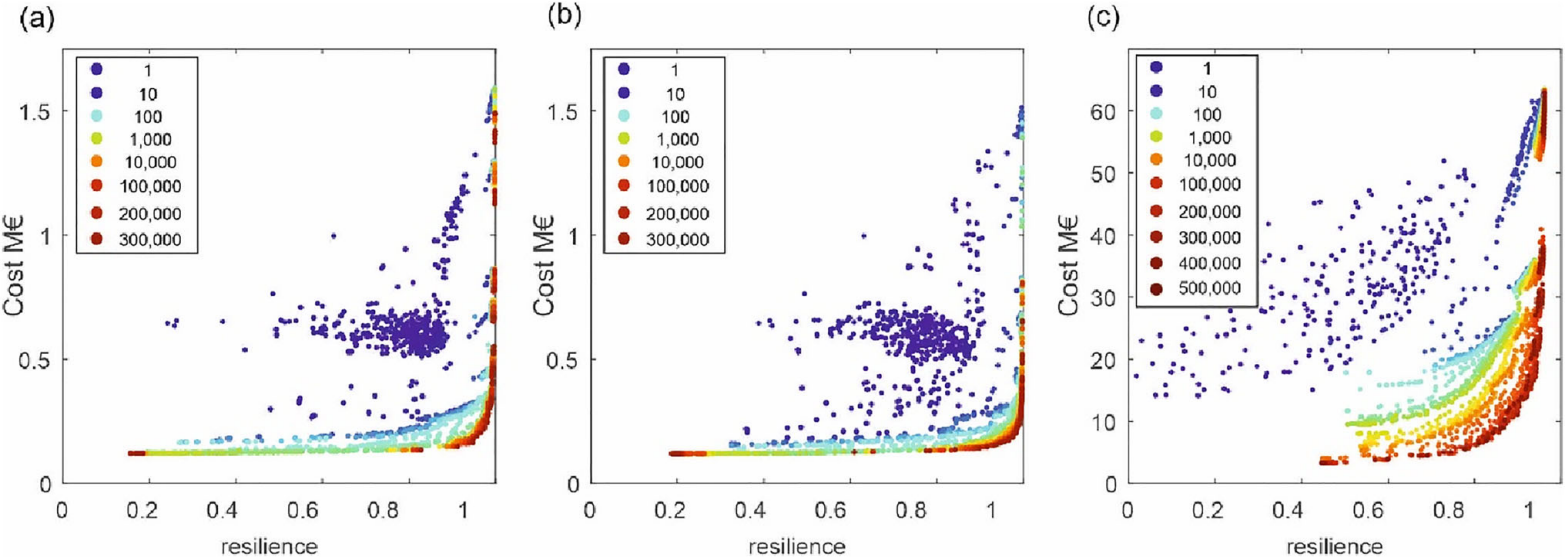
Results of the evolutionary optimization process to obtain Pareto-optimal WDN considering costs and resilience, **a** for one run of the small case study (118 demand nodes); **b** for the small cases study (10 demand nodes); **c** for the large case study

For the multi-objective optimization of the small real WDN, 300,000 generations and for the large one, 500,000 in the evolutionary algorithm were created and assessed. Due to the huge search space, it is difficult to determine an optimal population size. Based on numerical experiments and as a good balance between required computational time and a wide range of possible solutions [20] for population size, 100 individuals are used in each generation. To gain more confidence in the results of the evolutionary optimization, for the small case study with 118 demand nodes, the experiment was repeated 10 times with random initial populations resulting in a total of 380 million simulation runs of the hydraulic solver. Out of all generations, 47 for the small case study and 49 for the large case are investigated in more detail (see also Fig. 3).

Subsequently, the different design solutions of each real WDN are dual mapped with the edge diameter as the generalization model. In Fig. 3, the solutions (i.e., Pareto fronts of the design solutions) of these generations are investigated in more detail, from the initialization of the evolutionary algorithms (generation 1) to the (final) Pareto front of solutions (generations of 300,000 and 500,000 for the small and large case studies, respectively). Each dot in Fig. 3 shows one design solution of a WDN with its according costs (million €) on the *y*-axis, and the network resilience *I*_r_ (−) on the *x*-axis. The colors of the nodes are according to their generation (100 individuals in each generation). Note that to determine the network resilience for each solution, detailed hydraulic simulations are required, solving a large set of nonlinear equations using newton iteration while determining the nodal heads and pipe flows based on conservation of energy and mass. With increasing resilience, also the cost increase. A detailed description of the optimization process can be found in Sitzenfrei et al. [20].

## Results and discussions

**Fig. 4:**
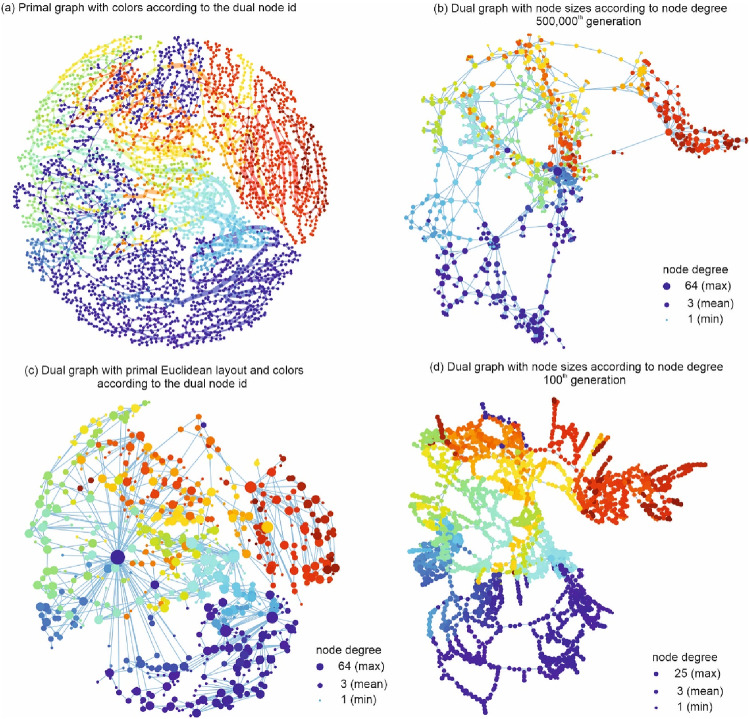
**a** Primal graph colors according to the dual nodes; **b** dual graph of a design solution of the 500,000th generation with marker sizes according to nodal degree; **c** dual graph with (mean) Euclidean coordinates for the dual graphs; **d** dual graph of a design solution from 100th generation with marker sizes according to nodal degree

In a first step, the dual characteristics of specific design solutions of the Pareto fronts are investigated to better outline the process of dual mapping of optimal design solutions. For that, in Fig. 4a, the primal graph of one single design solution of the large WDN is shown. This dual graph is part of the 500,000th (final) generation and has in total 878 dual nodes and 1326 dual edges. The resilience value *I*_r_ of that solution is 0.785 and the costs are 5.85 million € (see also Fig. 3). The line width of the edges corresponds to the real diameters, and the colors of the edges and the vertices correspond to the order of dual nodes found.

Figure 4b shows the dual graph of the same WDN with force-directed element placement and marker sizes according to the node degrees. The mean node degree in this dual graph is 3.02 with a maximum node degree of 64. The colours of the vertices are according to the order of dual nodes found. The used colors in Fig. 4b have the same order as in Fig. 4a. However, the spatial allocation between Fig. 4a, b is difficult to determine.

To approximately preserve the spatial location of the components and to better understand how the generalization model reduced the complexity of the primal graph (e.g., for detailed analysis or discussions), the spatial location is now considered for plotting the dual graph. Therefore, in Fig. 4c, the median Euclidean coordinates of the nodes in the primal graph, being part of a dual node, are used as new coordinates for the spatial layout of the dual graph. It can be seen that the node with the highest degree indeed connects many nodes from many regions of the network. This demonstrates that the edges integrated into this dual node can be viewed as a connector (connecting and transporting flows to many parts of the network), whose existence is of great importance for the functioning for the entire system. That dual node has the minimum available diameter (76.2 mm) of the design process for the generalization. More than 74% (2987 edges) of the pipes have that diameter in the primal network, 1017 of which are integrated into that dual node with a total pipe length of 58.48 km. The sum of total head loss in that dual node is 342.41 m (of in total 1625 m in the entire network), and the median flow in these edges is 1.04 L/s (maximum 1886.7 L/s in the entire network). This means that these edges with low diameter are responsible for 21% of the head losses, while only 0.05% of the maximum flow is transported.

As a first indicator of the differences in dual characteristics throughout the different generations, a dual graph of a design solution from an early generation is now exemplary investigated in Fig. 4d. The dual graph is again plotted with force-directed element placement and marker sizes according to node degree. The mean node degree in this dual graph is 3.18, which is almost the same as the network extracted from the 500,000th generation but with a maximum node degree of 25. This implies that the design solution taken from the earlier stages of optimization yielded much smaller maximum node degree. Similarly, the colour is based on the colors of the edges and the vertices is according to the order of dual nodes. In comparison to Fig. 4b, one can observe a much finely resolved structure, with lesser edges generalized into a dual node. This, indeed, indicates a heterogeneous formation of diameter distribution in the early stages of optimization, lessening the integration of identical edges into dual nodes.

To now gain a complete picture of the dual graph characteristics over the different generations and the populations within them, statistical analysis of the properties of all design solutions are investigated. Therefore, in the following, statistical evaluations of the entire multitude of investigated dual graphs are shown. By that we sought to answer the questions: how the dual characteristics change during the optimization process (i.e., with increasing generations) and are there some useful parameters that can describe how close a Pareto front is from an optimal solution?

**Fig. 5:**
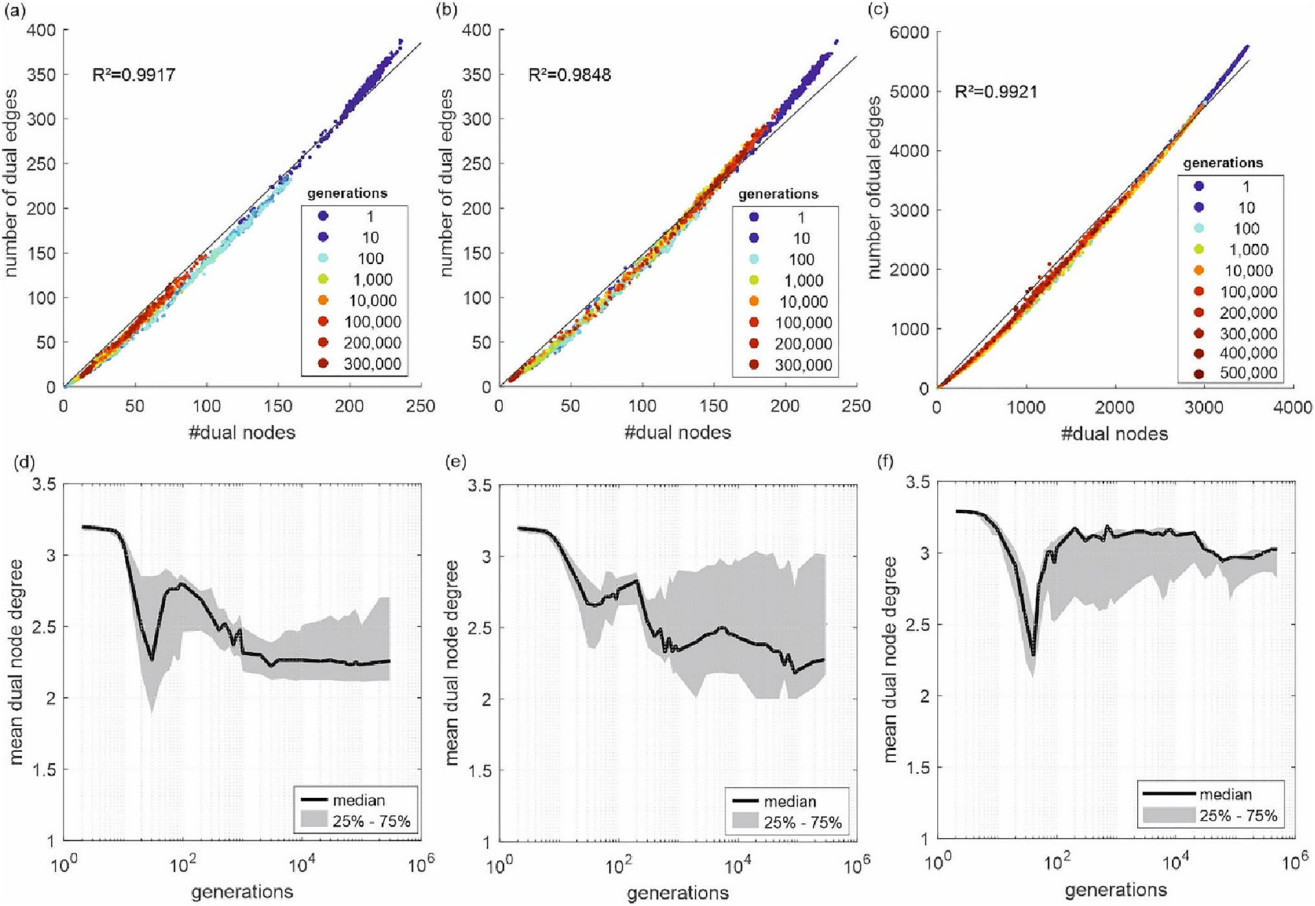
On the top row: plot of number of dual nodes (#dual nodes) against the number of dual edges (#dual edges) for one run of the small case study with 118 demand nodes (**a**), small case study with 10 demand nodes (**b**) and large case study (**c**). The colors of the dots indicate the generations in the optimization process. On the lower row: the mean dual node degrees (*y*-axis) are plotted against the number of generations (*x*-axis) for different case studies (**d**)–(**f**)

Therefore, in Fig. 5, the basic graph characteristics of in total 14,300 (47 + 47 + 49 generations with a population of 100 each) dual graphs are investigated. For the small case study (Fig. 5a), the number of dual nodes and edges continuously decreases with increasing the number of generations. While the average number of dual nodes for the first (randomly initialized) generation is around 216, it gets substantially reduced to 20, reaching the 300,000th generation. Notably, all dual mapped design solutions have an almost constant relationship between the number of dual nodes and the number of dual edges. A linear correlation results in a slope of the regression line of *k* = 1.54 with a coefficient equal to *R*^2^ = 0.9917. Although, for the more optimized solutions, this linear regression tends to overestimate the number of dual edges, while with the solutions with more randomness, the number of dual edges in comparison to the dual nodes is underestimated. A generation-wise linear fit produces relatively constant *k* = 1.48 for generations above 200, and *k*≈1.6 for the first generations.

**Fig. 6:**
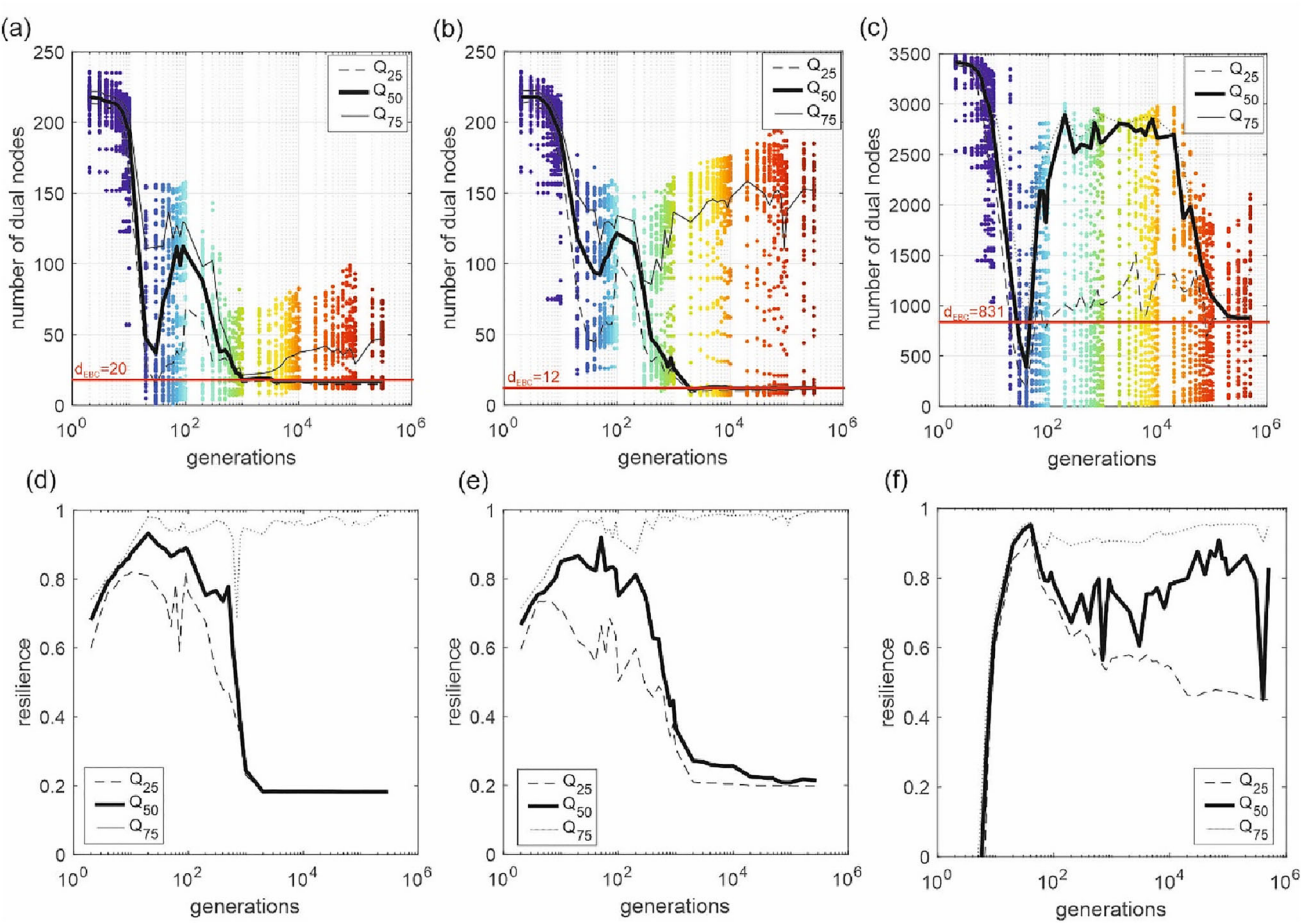
Number of dual nodes for different generations (colors) **a** small case study with 118 demand nodes; **b** small case study with 10 demand nodes; **c** large case study; **d**–**f** distribution of resilience values in the generations for different cases

Comparing these characteristics with those of the other case studies, a similar behavior can also be observed for the mean dual node degree for all the solutions within all the generations for the small case study (Fig. 5d). For the random initialization, the median node degree is 3.19 (almost no variation). With progressing optimization, the mean node degree sharply declines at the beginning, whereas it starts to rise/recover from generation 30 to 100. Within this region, the search algorithm in GALAXY appeared to have certain problems preserving the entire range of design solutions with respect to resilience (see also Fig. 6). These results could be of interest for further enhancing the capabilities of search operators.

For the small case study with a smaller number of demand nodes (Fig. 5b), the linear correlation analysis results in a slope of the regression line of *k* = 1.48 and *R*^2^ = 0.9848. A generation-wise linear fit produces relatively constant *k* = 1.50 for generations above 200 and *k*≈1.6 for the first generations.

A similar pattern can be observed for the large case study (Fig. 5c). The number of average dual nodes is decreasing from the initial population with an average of 3360 to 831 in the final generation. Analogous to the both small case studies, there exists an almost a linear correlation between the number of dual nodes and the number of dual edges for the large case study with a slope of the regression line of 1.58 and *R*^2^ = 0.992 (see Fig. 5c). However, again a slight nonlinear trend can be observed with less dual edges for more optimized solutions (higher number of generations). A generation-wise linear fit produces relatively constant *k* = 1.35 for generations above 60,000 and *k*≈1.6 for the generations before.

For the mean dual node degrees of the design solutions of the large case study (Fig. 5f), the behavior until the 100th generation shows a similar pattern compared to both small case studies (i.e., a sharp decline in the first generations, and then a quick recovery). However, thereafter a plateau is formed, lasting approximately until generation 10,000. One could now hypothesize that this plateau is also present for the small case study, but with much less extent (only until generation 300). After the plateau period, also the median dual node degree for the large case study decreases to 2.97. However, this drop is not as significant as for both small case studies. As a result, our study reveals that all case studies observed a sharp drop in the dual node degrees after the first generations followed by a plateau zone during the last stages of optimization (see also the interquartile ranges, specifically, the formation of a large interval shown in Fig. 5e. This explains that the mean degrees might be remaining constant in the upper territories). This implication indicates that the mean dual node degrees tend to remain constant after around the 1000 generations.

In Fig. 5a-c, the approximately linear relationship between dual nodes and dual edges was overserved. However, it has been observed that lower generations (dark blue dots) tend to be on the upper limit of dual elements, while the more progressed solutions (e.g., dark red dots) tend to be at the minimum number of dual elements. Therefore, the number of dual elements in dependence of the generations, specifically of the dual nodes, is now investigated in detail in the following to see if this characteristic can be used as indicators for the progress of the optimization.

To systematically analyze the dual characteristics in dependency of the generations, in Fig. 6a–c, the number of dual nodes (*y*-axis) against the number of generations (*x*-axis) for three case studies throughout the optimization process is plotted. For each generation, the entire population (all 100 solutions) is shown. The colors of the nodes are according to the generations. For statistical insight, in addition, the median value (*Q*_50_), the 25% (*Q*_25_), and 75% (*Q*_75_) percentiles are plotted to account for the uncertainties around the number of dual nodes. For the interpretation of the progress of the optimization, the ranges of the resilience values are also important. E.g., at the random initialization of the optimization process a very small range of the resilience values is present (see also Fig. 3, dark blue markers). Therefore, in Fig. 6d–f, the median (*Q*_50_), the 25% (*Q*_25_), and the 75% (*Q*_75_) percentiles of the resilience ranges for the different generations are plotted. A narrow range of resilience values for the initialization process can be observed for the first generation for the small case studies (Fig. 6a, b), while for the large case study, negative values are obtained for the initial generation (technically not feasible solutions, and not fulfilling the pressure constraints).

When having a closer look at Fig. 6a, one can observe that a formation pattern is almost analogous to the patterns of dual node degree in all generations; however, the number of dual nodes largely wanes at the tail of optimization despite some fluctuations in between. For the random initialization, the number of dual nodes is a little less than the number of decision variables (edges). This means that occasionally, two or more adjacent edges were found to have the same diameter and therefore, integrated into a dual node. Subsequently, there is a continuous decline in the number of dual nodes until generation 30, and the resilience value range of the solutions covers higher values. In this first period of the optimization process, the resilience of the solutions is continuously increasing, while low resilience solutions have not been found yet. For the 30th generation of the small case study, also the interquartile range of the resilience values of the design solutions is very small and between 0.81 and 0.98 (for example, from the 2000th generation on it is between 0.18 and 0.93) (see Fig. 6d). This pinpoints again that the search operators of the used evolutionary algorithm have certain troubles preserving/ensuring a wide range of technically feasible solutions during that stage of the optimization process. However, after the 30th generation, also the low resilient solutions are exploited, achieving a wide range of resilience values. Interestingly, the median number of dual nodes in Fig. 6a drops from 230 for the initial generation to approximately a constant value of 20 after generation 10,000. Furthermore, the resilience range in Fig. 6d has also its full extent and is not changing anymore. When having a closer look at Pareto fronts as well in Fig. 3a, one can notice that beyond generation 10,000, there is hardly any progress in the optimization process anymore. Therefore, the number minimal number of dual nodes could be an interesting indicator to assess the progress of the optimization process. While such an indicator could be calculated during the optimization itself, it would be even more beneficial to tell the approximate minimal number of dual nodes at the beginning of the process (i.e., based on the topology and the demand distribution). Based on the demand edge betweenness centrality, EBC(*k*), one could develop such an indicator. As described in the “Methods” section, with *d*_EBC_ it can be estimated, how many diameter changes are necessary with a given demand distribution. Therefore, in the following, the *d*_EBC_ values are evaluated and compared with the minimal numbers of dual nodes.

The red straight lines in Fig. 6a–c indicate the number of flow classes in the *d*_EBC_ values (calculated based on Eq. 4). For the small case study, the *d*_EBC_ is 20 which is very close to the median number of dual nodes in the design solutions from the higher generations. This means after the d_EBC_ value is reached, it is just a back and forth changing of diameters without any further progress in the optimization process. This is also supported by Fig. 3, where no significant changes from that generation further on can be observed.

The indicator *d*_EBC_ is based on the demand edge betweenness centrality and reflects the demand distribution within the WDN. To prove that the identified indicator works also for a different demand distribution, the small case study but with only 10 demand points is now investigated regarding minimal number of dual nodes and *d*_EBC_ (Fig. 6b). In general, almost similar behavior can be observed for the small case study with only 10 demand points (Fig. 6b). Notably, the number of *d*_EBC_ decreases to 12 while having less demand nodes. The median number of dual nodes during the middle generations (i.e., after 10,000th generations) converges again towards *d*_EBC_. This gives a strong indication that *d*_EBC_ gives an estimation of the expected minimal number of dual nodes during the evolutionary optimization.

As a next step, the correlation between *d*_EBC_ and the minimal number of dual nodes is investigated for the highly complex large case study. In principle, the same behavior can also be observed for the large case study (Fig. 6c). At the beginning of the optimization process (until the sixth generation), the resilience values are negative (technically not feasible solutions with pressure violations). After exploiting the high resilient solutions until the 40th generation, there is a rise in the number of dual nodes again while augmenting the resilience range of the design solutions (Fig. 6f). After a plateau of the mean number of dual nodes until the 10,000th generation, there is similarly a continuous decline. Most interestingly again, the minimal number of dual nodes converges towards *d*_EBC_ of 831, meeting each other between the 400,000th and 500,000th generations. In combination with the results from the two scenarios of the small case studies, it strongly indicates that from that point on there are no further changes expected regarding the mean number of dual nodes. Therefore, this territory can be regarded as a measure from which the final stage of the optimization process is achieved. Note that for the 500,000th generations, the optimization with GALAXY already took 35 weeks of computation time, indicating that no further changes are expected, and therefore, one can save a significant amount of further computation time.

Any evolutionary optimization process starts with some random initialization of the population. This implies that for different random starting points, the optimization could take different solutions paths. To gain better confidence in the obtained results, the experiments and evaluations are now repeated 10 times for the small case study with 118 demand nodes to see whether the conclusion that *d*_EBC_ is a good indicator for the expected minimal number of dual nodes in the optimization process still holds.

**Fig. 7:**
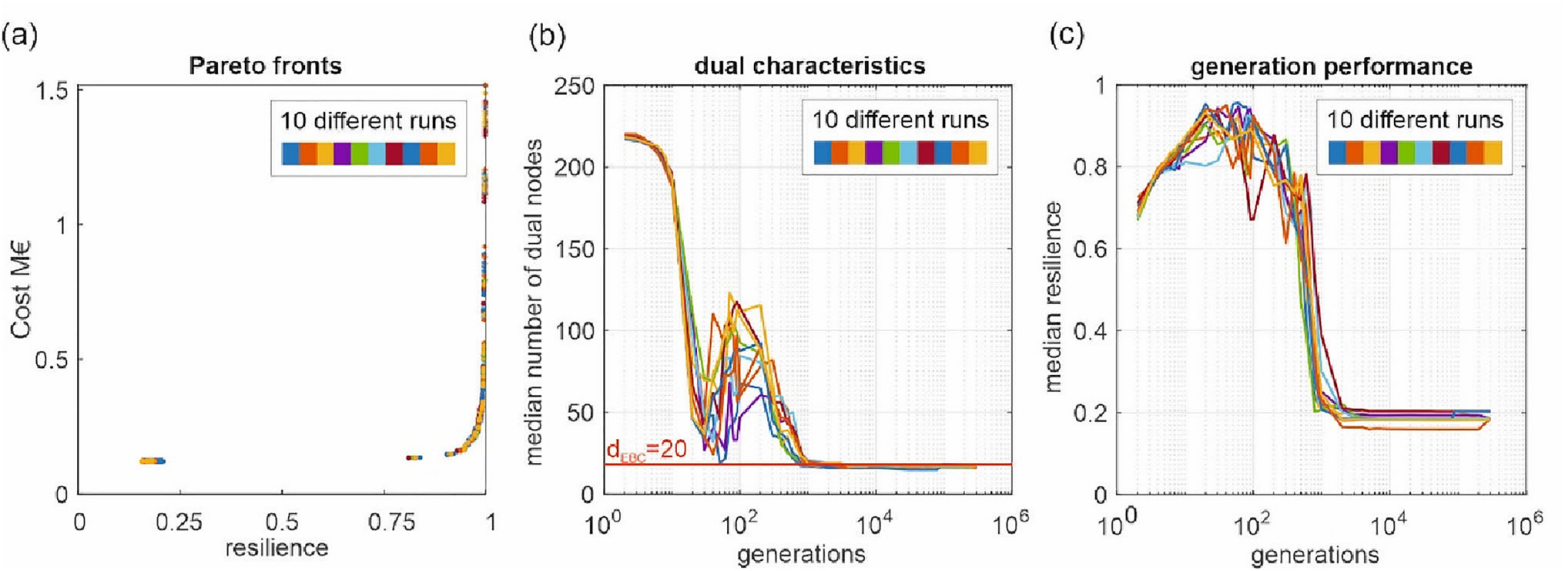
Results for 10 runs of the evolutionary optimization engine for the small case study with 118 demand nodes **a** Pareto fronts for the final generations (300,000th); **b** median values for the number of dual nodes in each generation; **c** median of resilience values in generations

In Fig. 7a, ten Pareto fronts of the final generation (300,000th) of the independent runs of GALAXY are shown. From a visual comparison, hardly any differences in the quality of the Pareto-fronts can be identified. This indicates that no further improvements can be expected with the given evolutionary algorithm and the chosen parameters. Note that GALAXY was conceptualized to optimize WDNs with only a minimum number of parameters which are the population size and the number of generations [26]. From additional numerical tests with a higher number of population sizes (up to 1000), no changes in the quality of the final generation were observed. Nevertheless, a significant additional computational burden was required. However, due to clarity of this manuscript, these results are not shown here, as they do not shed new light on the matter. In Fig. 7b, the median number of dual nodes during the different generations is investigated and compared to the *d*_EBC_ value. The behavior of the ten simulation runs fully supports the conclusion that the median number of dual nodes reaches a minimal value, and after that no further improvements in the optimization can be expected. Again, most interestingly, *d*_EBC_ gives, before the optimization starts, a target value for the minimal number of dual nodes. For completeness, in Fig. 7c, again the median resilience values of the different generations are shown (without the 25% and 75% percentile for better clarity). It can also be concluded from Fig. 6 that the resilience values do not change anymore beyond a certain generation (in this case approximately 10,000).

From the analysis shown in Figs. 6 and 7, it is inferred that 10,000 generations would be enough for the small case study to achieve optimal solutions. Although one could not assess the 10,000 at the beginning, with *d*_EBC_ representing the topology and the demand distribution, one has an estimate of the median of the minimal number of dual nodes before the optimization starts. Thus, in the optimization process, one can observe the number of dual nodes in a generation and decide whether more generations should be run with the evolutionary optimization engine. As the Pareto-front of that case study should have similar patterns under other evolutionary algorithms, the proposed approach of estimating *d*_EBC_ and comparing the number of dual nodes of the design solutions is also applicable to another evolutionary optimization of WDNs.

## Summary and conclusions

Often it seems that evolutionary algorithms for optimization can produce better results but with far more computational demands. However, how much time is needed to gain an optimal solution is difficult to answer.

In this work, for three different real case studies, the dual characteristics of 100 Pareto-optimal WDNs, including the pathway for optimization from random initialization to Pareto-optimal solutions are investigated (in total 14,300 design solutions). The optimization was performed with a multi-objective design approach, running up to 500,000 generations with a population size of 100 individuals for each case study. Two questions are investigated: (1) what are the dual graph characteristics of optimal WDNs, and (2) when is a sufficient optimization stage achieved, and how can that be assessed?

To identify the differences of optimal and non-optimal design solutions for WDNs, a dual graph approach that integrates the diameter as a generalization model was developed. Creating 14,300 dual graphs, the dual characteristics are systematically investigated to identify properties of optimal WDNs and verify whether dual characteristics can be used as an indicator of how close to optimal solutions the current solutions are.

It was found that the closer to optimal solutions, the less the number of dual nodes in the dual graphs. The minimum possible number of dual nodes is driven by the demand distribution of the WDNs. It was found that the flow classes determined with demand edge betweenness centrality (*d*_EBC_), give a good indicator for the minimal achievable mean dual node degree in the optimization process. Therefore, dual representation of the primal graph can be used as an indicator to assess if the evolutionary optimization process can still provide better results.

Therefore, the number of dual nodes with the minimum achievable value according to the characteristics of the demand distribution (*d*_EBC_) can be seen as an indicator of how far the optimization process proceeded and can be implemented to evolutionary optimization of WDNs to improve the procedure.

The proposed method does not require substantial additional computational burden and could be implemented to any meta-heuristic search engine used for optimization of water distribution networks. However, when introducing other objectives to the multi-objective optimization, which are less dependent on the network topology and the demand distribution, the indicator *d*_EBC_ might be less effective, and therefore, further research is required. The dual mapping approach might also be of interest for investigating the effectiveness of the search operators during the optimization approach. In this work, the range of solutions was quite narrow for some generations at the beginning of the optimization process. In this regard, future research could focus on systematically investigating the efficiency of search operators with the proposed dual approach.

## Data Availability

Water distribution networks as critical infrastructure are highly protected data. Therefore, the data and results of the networks are available in anonymized form via https://www.uibk.ac.at/umwelttechnik/softwareanddatasets/index.html.en.
